# Gestational age at birth and hospitalisations for infections among individuals aged 0–50 years in Norway: a longitudinal, register-based, cohort study

**DOI:** 10.1016/j.eclinm.2023.102108

**Published:** 2023-07-20

**Authors:** Sara Marie Nilsen, Jonas Valand, Tormod Rogne, Andreas Asheim, Weiyao Yin, Johanna Metsälä, Signe Opdahl, Henrik Døllner, Jan K. Damås, Eero Kajantie, Erik Solligård, Sven Sandin, Kari Risnes

**Affiliations:** a Center for Health Care Improvement, St. Olav's University Hospital, Norway; b Department of Clinical and Molecular Medicine, Norwegian University of Science and Technology, Trondheim, Norway; c Department of Chronic Disease Epidemiology, Yale School of Public Health, New Haven, CT, USA; d Center for Perinatal, Pediatric and Environmental Epidemiology, Yale School of Public Health, New Haven, CT, USA; e Department of Circulation and Medical Imaging, NTNU, Norwegian University of Science and Technology, Trondheim, Norway; f Department of Mathematical Sciences, Norwegian University of Science and Technology, Trondheim, Norway; g Department of Medical Epidemiology and Biostatistics, Karolinska Institutet, Stockholm, Sweden; h Department of Public Health and Welfare, Population Health Unit, Finnish Institute for Health and Welfare, Helsinki, Finland; i Department of Public Health and Nursing, Norwegian University of Science and Technology, Trondheim, Norway; j Children's Clinic, St. Olav's University Hospital, Trondheim, Norway; k Clinic of Medicine, Department of Infectious Diseases, St Olav's University Hospital, Trondheim, Norway; l Clinical Medicine Research Unit, Oulu University Hospital and University of Oulu, Oulu, Finland; m Department of Research and Development, Møre and Romsdal Hospital Trust, Ålesund, Norway; n Department of Psychiatry, Icahn School of Medicine at Mount Sinai, New York, USA; o Seaver Center for Autism Research and Treatment, Icahn School of Medicine at Mount Sinai, New York, NY, USA

**Keywords:** Gestational age, Preterm, Infectious disease, Respiratory tract infection, Hospitalisation risk

## Abstract

**Background:**

Preterm birth is associated with increased risk of childhood infections. Whether this risk persists into adulthood is unknown and limited information is available on risk patterns across the full range of gestational ages.

**Methods:**

In this longitudinal, register-based, cohort study, we linked individual-level data on all individuals born in Norway (January 01, 1967–December 31, 2016) to nationwide hospital data (January 01, 2008–December 31, 2017). Gestational age was categorised as 23–27, 28–31, 32–33, 34–36, 37–38, 39–41, and 42–44 completed weeks. The analyses were stratified by age at follow-up: 0–11 months and 1–5, 6–14, 15–29, and 30–50 years. The primary outcome was hospitalisation due to any infectious disease, with major infectious disease groups as secondary outcomes. Adjusted hospitalisation rate ratios (RRs) for any infection and infectious disease groups were estimated using negative binomial regression. Models were adjusted for year of birth, maternal age at birth, parity, and sex, and included an offset parameter adjusted for person-time at risk.

**Findings:**

Among 2,695,830 individuals with 313,940 hospitalisations for infections, we found a pattern of higher hospitalisation risk in lower gestational age groups, which was the strongest in childhood but still evident in adulthood. Comparing those born very preterm (28–31) and late preterm (34–36) to full-term (39–41 weeks), RRs (95% confidence interval) for hospitalisation for any infectious disease at ages 1–5 were 3.3 (3.0–3.7) and 1.7 (1.6–1.8), respectively. At 30–50 years, the corresponding estimates were 1.4 (1.2–1.7) and 1.2 (1.1–1.3). The patterns were similar for the infectious disease groups, including bacterial and viral infections, respiratory tract infections (RTIs), and infections not attributable to RTIs.

**Interpretation:**

Increasing risk of hospitalisations for infections in lower gestational age groups was most prominent in children but still evident in adolescents and adults. Possible mechanisms and groups that could benefit from vaccinations and other prevention strategies should be investigated.

**Funding:**

10.13039/501100011769St. Olav's University Hospital and 10.13039/100009123Norwegian University of Science and Technology, 10.13039/501100005416Norwegian Research Council, Liaison Committee for education, research and innovation in Central Norway, European Commission, Academy of Finland, 10.13039/501100006306Sigrid Jusélius Foundation, 10.13039/501100005744Foundation for Pediatric Research, and 10.13039/501100004325Signe and Ane Gyllenberg Foundation.

**Research in context**

**Evidence before this study**

Birth before full-term is associated with higher infection risk in childhood, but it is not known whether this risk persists in adulthood. We searched PubMed using the keywords (“gestational age” OR “preterm”) AND “infection” AND (“admission” OR “hospitalization” OR “hospitalisation” OR “inpatient”) for articles in any language from its inception up to October 19, 2022. The search returned 1592 articles, of which 15 concerned admissions due to infectious diseases among infants and children. We did not identify any studies on gestational age related to hospitalisations for infectious disease among adults.

**Added value of this study**

The risk of admissions due to infections increased gradually with lower gestational age. The increased risk was most pronounced in childhood but persisted into adulthood, although the estimated gap between those born preterm and full-term narrowed with increasing age. Higher hospitalisation rates in adults born preterm were evident for any infection and for both respiratory tract infections and infections not related to the respiratory tract.

**Implications of all the available evidence**

Birth before full term is a risk factor for infections in childhood, adolescence, and adulthood. Future studies should explore potential mechanisms including the roles of early life development of the microbiome, the immune system, and respiratory organ function. Preventive measures like vaccination strategies should be tailored to high-risk preterm born. Meanwhile, current knowledge may be used to optimize hygiene habits and vaccination guidelines, and to reduce known modifiable risk factors for severe infections such as smoking and obesity.

## Introduction

Host factors, including modifiable factors and genes, are increasingly recognised as important risk factors and targets for the prevention of infections.^1^^,^^2^ Although the development of immune responses is modulated by early life exposure, the role of perinatal factors in long-term infection risk is understudied. Preterm birth, defined by the World Health Organization (WHO) as a gestation shorter than 37 completed weeks, is a strong predictor of mortality and chronic disease^3^, ^4^, ^5^; but its role in the life-long risk of infectious diseases remains unestablished.

Worldwide, 15 million babies are born preterm every year, and complications following preterm birth are the leading cause of death in children aged <5 years, causing 1 million annual deaths.^6^ The high burden of neonatal and childhood morbidity and mortality in preterm births has been extensively reported,^7^ with the highest risk in the most preterm group. Increasingly, studies point to excess long-term risk for non-communicable diseases in individuals born late preterm (34–36 weeks) and early term (37–38 weeks).^3^^,^^8^, ^9^, ^10^ The early term group constitutes approximately 20% of all births, underlining public health concerns.

Recently, infections were identified as the primary cause of excess hospital contacts in children born preterm in the UK, pointing to a research priority on preventing infections in this group.^11^ A registry linkage study from Australia found an inverse association between gestational age and the risk of hospitalisation due to infection that declined with increasing age, but persisted in adolescents up to 18 years.^12^ However, a Danish nation-wide register study found that the initially higher risk of hospitalisations related to RTIs in preterm children gradually decreased with age and ultimately disappeared during adolescence.^13^ Thus, it remains uncertain whether preterm born may outgrow their susceptibility to infections by adulthood. However, there are biological cues to the role of gestational age at birth in altering long-term infection risk. The immune system starts to develop *in utero*, and immune responses continue to develop after birth and are influenced by environmental exposure, which may differ according to the length of gestation. Thus, birth before full term may disturb the long-term immune response to infection.

Given the known link between preterm birth, alterations in the immune system and childhood infections, we hypothesized that this association persists into adulthood. This study aimed to determine hospitalisation rates for infectious diseases at different ages, from infancy up to 50 years, by comparing individuals born at different gestational ages.

## Methods

### Data sources and study design

We used data from the Medical Birth Registry of Norway (MBRN), which contains complete coverage of all live and still births in Norway since 1967.^14^^,^^15^ Records of all births from 1967 to 2016 were linked to data from the Norwegian Patient Registry (NPR) on all infection-related hospitalisations, including national data from the start of personal identification in NPR in 2008 and through 2017.^16^ All Norwegian hospitals have since 2008 submitted information about their clinical activity to NPR.^16^ Data from NPR is routinely analyzed by the National Service for Validation and Completeness Analyses, and in general NPR data have a high level of completeness.^16^ The unique 11-digit identity number that is allocated to every Norwegian resident at birth or immigration makes it possible to link data from several sources. Data on maternal educational level at the time of birth were available from Statistics Norway. The date of death and date of emigration were available from the Norwegian Cause of Death Registry,^17^^,^^18^ and the registry's degree of coverage is near-complete.^19^ The population was defined by the MBRN, where a project-specific participant identifier was created. The researchers only had access to de-identified data. The study was conducted following the guidelines outlined in the Strengthening the Reporting of Observational Studies in Epidemiology (STROBE) checklist.

The study and data linkage were approved by the Regional Committee for Medical and Health Research (Rek-Midt 2018/32) and the registry-keeping authorities.

### Study population

All individuals born in Norway from 1967 and through 2016 were eligible. Individuals were excluded if their identification number was missing; gestational age was outside a range which would typically have been actively treated (for birth years ≤1987: 26–44 weeks, >1987: 23–44 weeks); stillborn; birthweight missing. To reduce the impact of incorrect measures of exposure, we excluded individuals with a birthweight of >6000 g or <350 g, a birthweight for gestational age standard deviation (SD) score >4 or < −6 from expected or maternal age >55 years.^20^ We also excluded those who died or emigrated before the start of follow-up in 2008. Although obtaining informed consent is neither mandatory nor practiced for inclusion in the national registries utilized in this study, individuals have the right to object to the inclusion of specific data in the MBRN.

### Gestational age

Information on gestational age in days was obtained from the MBRN. Gestational age was estimated based on ultrasound examination or the last menstrual period, if ultrasound was not performed. In the case of assisted reproduction, estimation from embryo transfer was preferred over ultrasonography. Gestational age in days was converted to completed weeks at birth and categorized as extremely preterm (23–27), very preterm (28–31), moderately preterm (32–33), late preterm (34–36), early term (37–38), full-term (39–41), and post-term (42–44 weeks) and gestational age was also treated as a continuous variable based on completed days of gestation.

### Outcomes

The primary outcome was inpatient hospitalisation for infectious diseases based on the International Classification of Diseases (ICD-10) discharge diagnoses assigned by specialists only and collected for administrative purposes from the NPR database. We also studied admissions due to respiratory tract infections (RTIs) and non-RTIs separately and any infection caused by specific viral or bacterial agents separately. Furthermore, bacterial and viral infection-related admissions were studied separately for RTIs and non-RTIs. All ICD-10-codes used to identify infections are listed in Supplemental Table S1. Only infections that were defined as the main diagnosis assigned at discharge were considered. NPR include complete data on hospital admissions in Norway from 2008 and defined the timeframe for follow-up when information regarding infection admissions (2008–2017) was possible. Consequently, this defined the relevant period within which individuals could potentially be at risk of admissions in the study.

### Statistical analyses

**Fig. 1:**
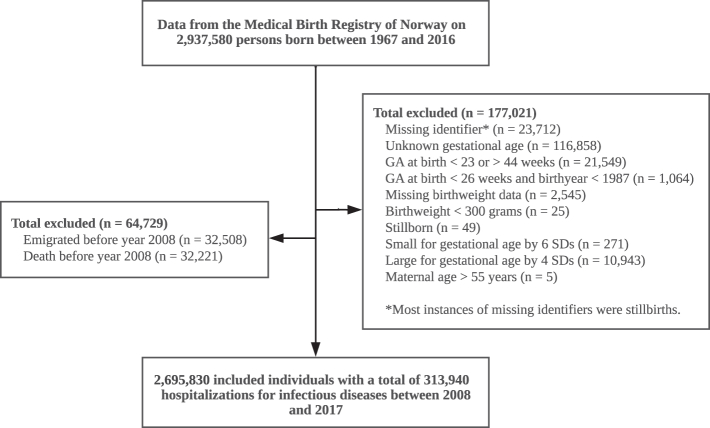
Flowchart. Successive exclusions.

Analyses were performed separately for five age groups: 0–11 months, 1–5 years, 6–14 years, 15–29 years, and 30–50 years. Associations between gestational age at birth and hospitalisation for infections were quantified by calculating the hospitalisation rates (number of inpatient hospital admissions per 1000 person-years) and hospitalisation rate ratios and comparing individuals born in different categories of gestational age. We calculated the person-time at risk for hospital admissions due to infectious disease from 2008 to 2017 that each individual contributed within their respective age-groups, and the corresponding number of hospitalisations. For example, an individual who was born on January 01, 1967 was considered at risk from January 01, 2008 through December 31, 2017 when counting hospitalisations in the age category of 30–50 years. We subtracted the time spent in hospital for an infection from the time at risk, as patients would not be at risk of having a new admission for infection during this period. Individuals did not contribute with person-time after emigration or death. Using this design, different birth-year cohorts were followed up at different ages (Supplemental Figure S1). Hospitalisation rates and rate ratios (RRs) were estimated using negative binomial regression models, one for each age group, with robust standard errors. Models were adjusted for year of birth, maternal age at birth, parity (firstborn or not), and sex and included an offset parameter adjusted for person-time at risk. We calculated the adjusted hospitalisation RRs and associated two-sided 95% confidence intervals (CIs) for gestational age categories and also estimated linear associations per 30 days longer gestational age. The negative binomial model was chosen because assumptions about the association between the mean and variance are less restrictive compared with Poisson regression.^21^ To assess whether effect measures were modified by sex, we fitted a negative binomial regression model including an interaction term between gestational age and sex. We found little evidence of any effect measure modification by sex on a multiplicative scale (p > 0.05; Supplemental Table S2) and presented all outcomes for men and women combined, adjusted for sex. Following exclusions as outlined in Fig. 1, we performed complete case analyses in the main analyses.

All statistical models were estimated using Stata version 17.0.^22^ Forest plots were created using Python and the plotting library Matplotlib.^23^

### Additional analyses

We evaluated consistency using additional analyses, adjusting for maternal educational level at birth, maternal country of birth, birthweight SD score and birth months in two categories of Spring–Summer (March–August) and Fall–Winter (September–February). These were complete case analyses. The number of individuals excluded in addition to the main analyses, are reported in relation to each figure in the addition analyses, and in Table 1. Additional analyses to assess robustness were also performed after excluding specific risk groups, one at a time: persons with congenital malformations (identified from MBRN), persons with CP/severe disability defined from any hospital contact with primary or secondary diagnosis F72, F73 or G80.0 between 2008 and 2017, and cases of multiple pregnancies.

**Table 1:** Characteristics of the study population, by gestational age at birth and age at follow-up.

	Gestational age at birth in weeks						
	23–27	28–31	32–33	34–36	37–38	39–41	42–44
**0–11 months**	n = 1409	n = 3750	n = 4974	n = 26,908	n = 106,616	n = 423,899	n = 28,819

Maternal age in years at birth (SD)	31 (6)	30 (6)	30 (6)	30 (6)	30 (5)	30 (5)	30 (5)
Nulliparous, n (%)	750 (53)	2120 (57)	2689 (54)	12,867 (48)	41,222 (39)	178,025 (42)	16,117 (56)
Males, n (%)	750 (53)	2032 (54)	2692 (54)	14,203 (53)	53,116 (50)	216,357 (51)	17,186 (60)
Mean birthweight in grams (SD)	828 (205)	1396 (323)	1954 (372)	2620 (458)	3217 (457)	3634 (448)	3900 (449)
Primary education, n (%)	346 (25)	787 (21)	1013 (20)	5279 (20)	20,454 (19)	71,540 (17)	4688 (16)
Education missing, n (%)	85 (6)	219 (6)	222 (4)	1294 (5)	5753 (5)	22,308 (5)	1905 (7)
Maternal birth country not Norway, n (%)	393 (28)	890 (24)	1153 (23)	6267 (23)	27,864 (26)	101,478 (24)	7075 (25)
Maternal birth country missing, n (%)	30 (2)	67 (2)	85 (2)	428 (2)	1531 (1)	6230 (1)	523 (2)
**1–5 years**	n = 1932	n = 5615	n = 7609	n = 40,345	n = 156,826	n = 609,122	n = 48,866

Maternal age in years at birth (SD)	30 (6)	30 (6)	30 (6)	30 (5)	30 (5)	30 (5)	30 (5)
Nulliparous, n (%)	1014 (52)	3134 (56)	4087 (54)	19,354 (48)	60,121 (38)	252,390 (41)	26,212 (54)
Males, n (%)	999 (52)	3012 (54)	4091 (54)	21,113 (52)	77,863 (50)	310,435 (51)	29,136 (60)
Mean birthweight in grams (SD)	844 (203)	1402 (328)	1966 (372)	2634 (464)	3234 (460)	3645 (453)	3916 (460)
Primary education, n (%)	445 (23)	1178 (21)	1536 (20)	7965 (20)	30,288 (19)	103,042 (17)	7879 (16)
Education missing, n (%)	113 (6)	280 (5)	340 (4)	1794 (4)	7950 (5)	29,731 (5)	2806 (6)
Maternal birth country not Norway, n (%)	472 (24)	1169 (21)	1519 (20)	8301 (21)	36,629 (23)	129,071 (21)	9936 (20)
Maternal birth country missing, n (%)	47 (2)	101 (2)	152 (2)	716 (2)	2518 (2)	9785 (2)	905 (2)
**6–14 years**	n = 2177	n = 6963	n = 9392	n = 48,919	n = 177,664	n = 719,748	n = 91,417

Maternal age in years at birth (SD)	30 (6)	30 (5)	30 (5)	29 (5)	30 (5)	29 (5)	29 (5)
Nulliparous, n (%)	1075 (49)	3755 (54)	4892 (52)	23,470 (48)	68,415 (39)	289,857 (40)	44,353 (49)
Males, n (%)	1118 (51)	3781 (54)	5078 (54)	26,019 (53)	90,267 (51)	365,724 (51)	50,317 (55)
Mean birthweight in grams (SD)	861 (202)	1409 (344)	1982 (392)	2666 (491)	3256 (470)	3658 (466)	3876 (490)
Primary education, n (%)	556 (26)	1700 (24)	2235 (24)	11,378 (23)	38,842 (22)	141,682 (20)	19,144 (21)
Education missing, n (%)	89 (4)	215 (3)	317 (3)	1432 (3)	6320 (4)	21,999 (3)	2707 (3)
Maternal birth country not Norway, n (%)	394 (18)	1031 (15)	1334 (14)	7226 (15)	30,157 (17)	102,392 (14)	11,078 (12)
Maternal birth country missing, n (%)	79 (4)	226 (3)	342 (4)	1553 (3)	5587 (3)	22,438 (3)	3242 (4)
**15–29 years**	n = 1663	n = 6561	n = 9374	n = 51,807	n = 175,496	n = 851,129	n = 157,432

Maternal age in years at birth (SD)	29 (5)	28 (5)	28 (5)	28 (5)	28 (5)	28 (5)	27 (5)
Nulliparous, n (%)	769 (46)	3337 (51)	4759 (51)	25,202 (49)	71,421 (41)	343,326 (40)	74,056 (47)
Males, n (%)	860 (52)	3606 (55)	5157 (55)	28,312 (55)	94,491 (54)	431,798 (51)	79,165 (50)
Mean birthweight in grams (SD)	900 (201)	1448 (364)	2014 (439)	2711 (530)	3249 (481)	3630 (474)	3777 (500)
Primary education, n (%)	527 (32)	2170 (33)	3105 (33)	16,772 (32)	52,849 (30)	245,624 (29)	50,694 (32)
Education missing, n (%)	43 (3)	215 (3)	340 (4)	1975 (4)	6182 (4)	28,722 (3)	6152 (4)
Maternal birth country not Norway, n (%)	185 (11)	582 (9)	780 (8)	4652 (9)	17,312 (10)	61,384 (7)	9190 (6)
Maternal birth country missing, n (%)	146 (9)	657 (10)	1027 (11)	5277 (10)	18,471 (11)	93,445 (11)	17,844 (11)
**30–50 years**	n = 428	n = 3728	n = 6292	n = 38,134	n = 128,881	n = 734,009	n = 150,471

Maternal age in years at birth (SD)	27 (6)	26 (6)	26 (6)	26 (6)	27 (6)	26 (5)	25 (5)
Nulliparous, n (%)	198 (46)	1826 (49)	3042 (48)	17,444 (46)	51,350 (40)	293,245 (40)	70,544 (47)
Males, n (%)	219 (51)	2041 (55)	3471 (55)	20,991 (55)	70,737 (55)	371,144 (51)	74,093 (49)
Mean birthweight in grams (SD)	1059 (199)	1562 (362)	2090 (448)	2748 (541)	3219 (483)	3581 (472)	3697 (493)
Primary education, n (%)	182 (43)	1534 (41)	2612 (42)	15,441 (40)	48,967 (38)	261,321 (36)	57,000 (38)
Education missing, n (%)	23 (5)	280 (8)	437 (7)	2642 (7)	7427 (6)	40,747 (6)	10,255 (7)
Maternal birth country not Norway, n (%)	19 (4)	122 (3)	233 (4)	1432 (4)	5077 (4)	22,254 (3)	3973 (3)
Maternal birth country missing, n (%)	98 (23)	826 (22)	1433 (23)	8762 (23)	29,482 (23)	166,397 (23)	33,622 (22)

### Role of the funding source

The funders of the study had no role in study design, data collection, data analysis, data interpretation, or writing of the report. Authors KR, JV and SMN had full access to all the data in the study and takes responsibility for the integrity of the data and the accuracy of the data analysis. All authors take final responsibility for the decision to submit the report for publication.

## Results

A total of 2,937,580 births were registered in Norway from 1967 to 2016. After all exclusions, the study population consisted of 2,695,830 individuals (92% of births during the study period; Fig. 1). The characteristics of the population by age at follow-up and gestational age category at birth are shown in Table 1. In the study population, 313,940 eligible hospitalisations occurred between 2008 and 2017, with infection defined as the main diagnosis and primary medical concern (Supplemental Table S3).

### Hospitalisations due to any infectious disease

**Fig. 2:**
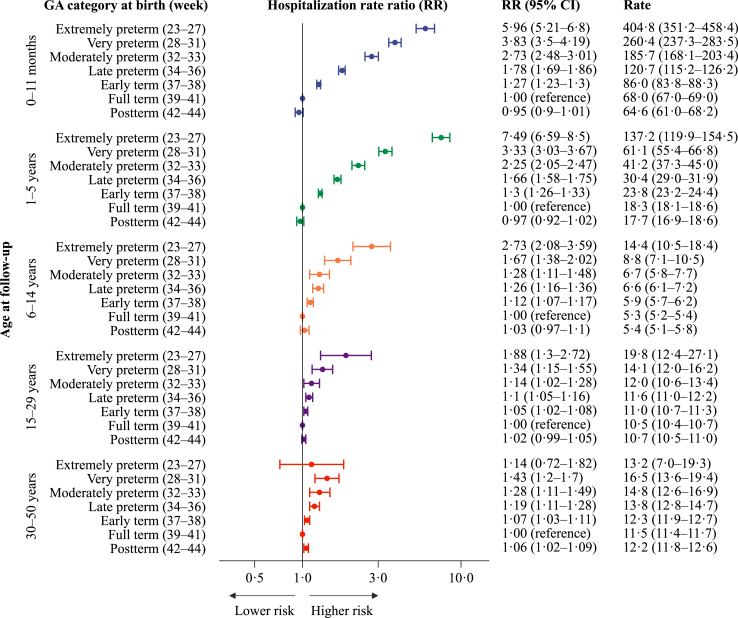
Estimated yearly rates (per 1000 person-years) and rate ratios for hospitalisations related to any infectious diseases (2008–2017), by gestational age (GA) at birth. Hospitalisation rate ratios (RR) are plotted on a logarithmic scale and were calculated with individuals born at term (week 39–41) as references. The error bars represent the 95% confidence intervals (95% CI) for the RRs. The results are adjusted for year of birth, maternal age, parity, and sex. There were 313,940 admissions for any infectious diseases.

Overall, hospitalisation rates per 1000 person-years for any infection were the highest during infancy (80.4), lowest at ages 6–14 years (5.5) and increased slightly into adulthood (11.8 at ages 30–50) (Supplemental Table S4). For all age groups, hospitalisation rates were highest in the lower gestational age groups and decreased gradually with increasing gestational age at birth up to the early term group (Fig. 2). A higher risk of hospitalisation with lower gestational age was most pronounced during infancy and early childhood. For those born very preterm (week 28–31) compared with those born at full-term, the adjusted hospitalisation RR (aRR) (95% CI) was 3.3 (3.0–3.7) at ages 1–5, and for late preterm birth (week 34–36), the corresponding estimate was 1.7 (1.6–1.8) at ages 1–5.

Among adults aged 30–50 years, hospitalisation rates were higher in the earlier categories of preterm birth. For very and late preterm compared with full-term, the aRRs (95% CIs) were 1.4 (1.2–1.7) and 1.2 (1.1–1.3), respectively. In this age group, the estimate was imprecise for those born extremely preterm. For any infectious disease hospitalisations, the aRRs per 30 days longer gestation were gradually less pronounced with older age groups, but remained evident in adulthood: 0.62 (0.61–0.63) at 1–5 years, 0.84 (0.81–0.87) at 6–14 years and 0.94 (0.91–0.97) at 30–50 years (Table 2).

**Table 2:** The risk of hospitalisations per 30 days longer gestational age (95% CI).

	Any infectious disease	Respiratory tract infections (RTIs)	Viral infectious diseases	Bacterial infectious diseases	Viral RTIs	Bacterial RTIs	Any infectious diseases not RTIs	Viral infectious diseases not RTIs	Bacterial infectious diseases not RTIs
0–11 months	0.61 (0.59–0.62)	0.50 (0.49–0.51)	0.53 (0.51–0.54)	0.71 (0.66–0.75)	0.49 (0.48–0.51)	0.51 (0.45–0.58)	0.85 (0.82–0.89)	0.74 (0.67–0.82)	0.75 (0.69–0.81)
1–5 years	0.62 (0.61–0.63)	0.55 (0.54–0.57)	0.52 (0.50–0.54)	0.70 (0.66–0.73)	0.48 (0.46–0.50)	0.59 (0.56–0.63)	0.74 (0.72–0.77)	0.72 (0.67–0.78)	0.80 (0.74–0.87)
6–14 years	0.84 (0.81–0.87)	0.70 (0.65–0.75)	0.81 (0.74–0.89)	0.81 (0.75–0.87)	0.67 (0.59–0.77)	0.68 (0.60–0.77)	0.91 (0.88–0.95)	0.92 (0.82–1.03)	0.87 (0.80–0.95)
15–29 years	0.93 (0.91–0.96)	0.87 (0.82–0.92)	0.98 (0.93–1.03)	0.87 (0.83–0.92)	0.83 (0.73–0.94)	0.86 (0.77–0.95)	0.95 (0.93–0.98)	1.01 (0.95–1.07)	0.88 (0.83–0.93)
30–50 years	0.94 (0.91–0.97)	0.90 (0.86–0.95)	0.95 (0.88–1.02)	0.91 (0.87–0.96)	0.87 (0.76–0.98)	0.90 (0.83–0.97)	0.95 (0.92–0.98)	0.99 (0.90–1.08)	0.93 (0.88–0.98)

### Hospitalisations due to respiratory tract infections

**Fig. 3:**
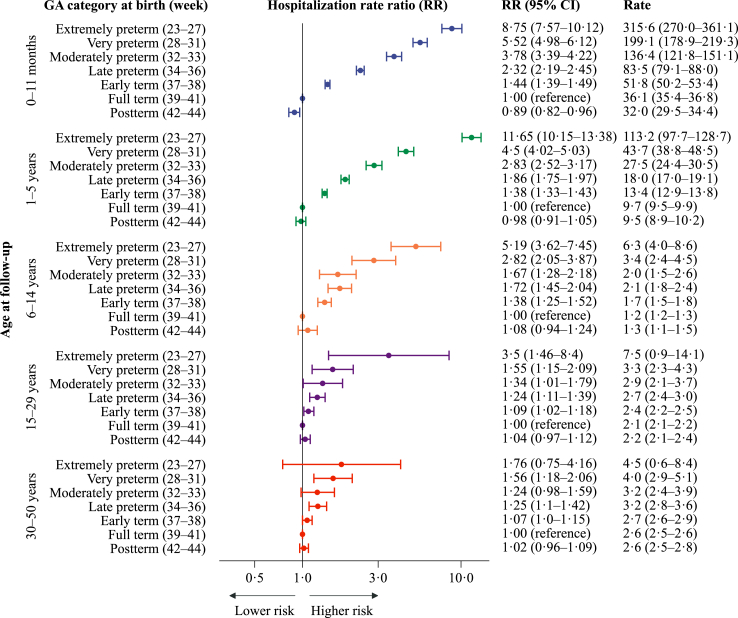
Estimated yearly rates (per 1000 person-years) and rate ratios for hospitalisations related to respiratory tract infections (2008–2017), by gestational age (GA) at birth. Hospitalisation rate ratios (RR) are plotted on a logarithmic scale and were calculated with individuals born at term (week 39–41) as references. The error bars represent the 95% confidence intervals (95% CI) for the RRs. The results are adjusted for year of birth, maternal age, parity, and sex. There were 107,380 admissions for respiratory tract infections.

One in three hospitalisations for infectious diseases were classified as RTIs (n = 107,380, Supplemental Table S5). In terms of the risk of hospitalisation for childhood infections, the excess risk of being preborn compared with full-term was more pronounced for RTIs than for other infections (Fig. 3). For RTIs at ages 1–5, the RRs (95% CI) for the very (week 28–31) and late preterm groups (week 34–36) compared with full-term were 4.5 (4.0–5.0) and 1.9 (1.8–2.0). A pattern of higher risk with lower gestational age was evident in all age groups; however, the estimates were less pronounced with increasing age. For RTIs at ages 30–50, the corresponding RRs (95% CIs) were 1.6 (1.2–2.1) and 1.3 (1.1–1.4). Of the 107,380 RTI hospitalisations, 22,294 (21%) were identified as being caused by viral and 21,180 (20%) by bacterial agents. The aRRs (95% CIs) per 30 days longer gestation were more pronounced for RTIs than for any infections: 0.55 (0.54–0.57) at 1–5 years, 0.70 (0.65–0.75) at 6–14 years and 0.90 (0.86–0.95) at 30–50 years (Table 2).

### Viral RTIs

Hospitalisation rates for viral RTIs were the highest at age 0–11 months (13 and 79 per 1000 person-years, respectively, in the full-term and extremely preterm groups), declining thereafter; among adults, there was <1 hospitalisation for viral RTIs per 1000 person-years (Supplemental Figure S2). Comparing the very and late preterm groups with term born at ages 1–5, the adjusted RRs (95% CIs) for viral RTIs were 7.4 (6.3–8.7) and 2.0 (1.8–2.3). For viral RTIs at ages 30–50, the corresponding estimates were 3.1 (1.7–5.6) and 1.3 (1.1–1.7).

### Bacterial RTIs

In adulthood, hospitalisation rates for bacterial RTIs were considerably higher than those for viral RTIs, and the patterns of associations for lower gestational age groups for bacterial RTIs were comparable but with an overall higher precision than that of viral RTIs (Supplemental Figure S3).

### Hospitalisations due to viral and bacterial infectious diseases

**Fig. 4:**
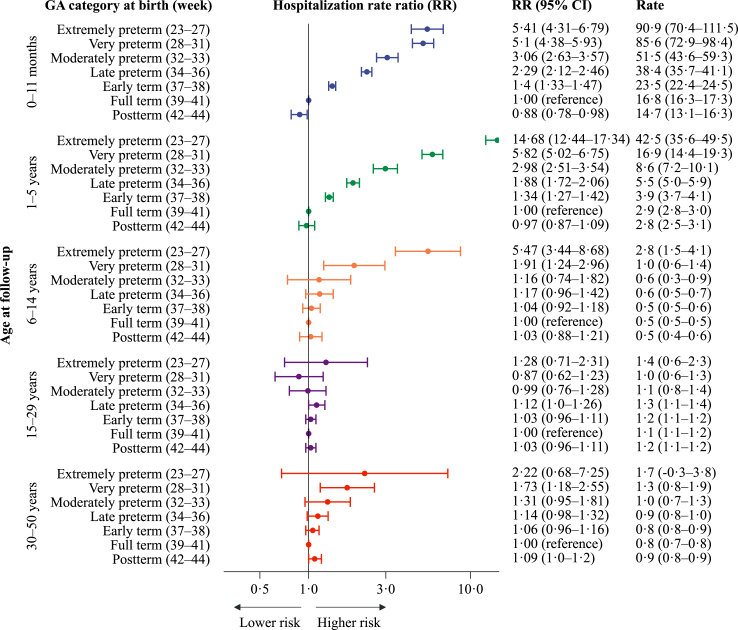
Estimated yearly rates (per 1000 person-years) and rate ratios for hospitalisations related to viral infectious diseases (2008–2017), by gestational age (GA) at birth. Hospitalisation rate ratios (RR) are plotted on a logarithmic scale and were calculated with individuals born at term (week 39–41) as references. The error bars represent the 95% confidence intervals (95% CI) for the RRs. The results are adjusted for year of birth, maternal age, parity, and sex. There were 41,067 admissions for viral infectious diseases.

**Fig. 5:**
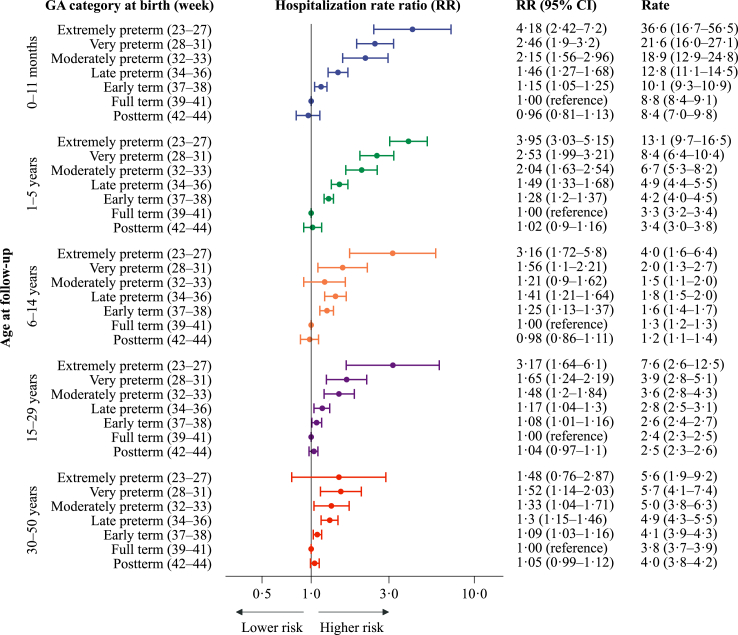
Estimated yearly rates (per 1000 person-years) and rate ratios for hospitalisations related to bacterial infectious diseases (2008–2017), by gestational age (GA) at birth. Hospitalisation rate ratios (RR) are plotted on a logarithmic scale and were calculated with individuals born at term (week 39–41) as references. The error bars represent the 95% confidence intervals (95% CI) for the hospitalisation rate ratios (RR). The results are adjusted for year of birth, maternal age, parity, and sex. There were 76,048 admissions for bacterial infectious diseases.

Overall, 41,067 (13.1%) hospitalisations due to infection could be attributed to viral agents (Supplemental Table S6) and 76,048 (24.2%) to bacterial agents (Supplemental Table S7). In childhood, estimated aRRs per 30 days longer gestation were more pronounced for viral than for bacterial diseases, while in adulthood, the corresponding estimates were more pronounced for bacterial than for viral diseases (Table 2). For viral infections, the estimated excess risks for the most preterm groups compared with full-term were particularly high during early childhood, whereas less pronounced patterns were noted from 6 to 14 years and beyond (Fig. 4). However, for bacterial infections, a pattern of higher RRs with lower gestational age was also evident in adolescents and adults (Fig. 5). Comparing the very and late preterm groups with term born at ages 1–5, the adjusted RRs (95% CIs) for bacterial infections were 2.5 (2.0–3.2) and 1.5 (1.3–1.7). At ages 30–50, the corresponding estimates were 1.5 (1.1–2.0) and 1.3 (1.2–1.5).

### Hospitalisations for infections that were not RTIs

For infections not classified as RTIs, the results also indicated a gradient pattern for gestational age that was attenuated by age but was still evident in adulthood, but with overall less pronounced estimates than for RTIs (Table 2 and Supplemental Figure S4). For viral infections that were not classified as RTIs, an increased risk in groups born before full term was evident in childhood but attenuated in adolescence and adulthood (Supplemental Figure S5). However, for bacterial infections excluding RTIs, the groups born before full term had a persistent increase in risk during childhood, adolescence, and adulthood, although estimates for individuals born extremely preterm lacked precision and were not stable across age groups at follow-up (Supplemental Figure S6). Comparing the very and late preterm groups with term born at ages 30–50, the adjusted RRs (95% CIs) for bacterial non-RTI infections were 1.5 (1.1–3.2) and 1.3 (1.1–1.5).

### Additional analyses

We adjusted for birthweight SD scores (Supplemental Figure S7), maternal education at birth (Supplemental Figure S8), maternal country of birth (Supplemental Figure S9) and season of birth (Supplemental Figure S10). None of these adjustments substantially changed the associations. When excluding 84,226 individuals with congenital malformations, 4375 individuals diagnosed with cerebral palsy or developmental disorders, and 75,519 multiples, the results were comparable to those of the entire cohort (Supplemental Figures S11–S13, respectively).

A summary of the estimates for the very preterm and late preterm groups for all infectious groups in early childhood (1–5 years) and adulthood (30–50 years) is shown in Supplemental Figure S14.

## Discussion

In this study of 2.7 million individuals, we found a gradual increase in infection-related hospitalisation risk with a lower gestational age at birth. The association was very strong in childhood, persisted throughout adolescence and adulthood, was robust to adjustment for perinatal and maternal factors, and could not be explained by congenital malformations or severe neurological conditions. In the very preterm group, there was three-fold increase in the risk of hospitalisation for any infection in childhood, whereas this association was attenuated to a 30–40% increased risk in the adult groups. At ages 30–50, the pattern of gradually increasing infection-related hospitalisation risk with lower gestational age at birth was most pronounced for bacterial and viral RTIs and for bacterial infections that were not RTIs.

Our study is the first to assess the risk of adult infection across the entire gestational age spectrum. Our finding that preterm birth is associated with increased risk of hospitalisation for infections in childhood corresponds well with those of previous studies, although most previous studies were typically follow-up studies of extremely preterm groups and not population-based. Studies on preterm children show an elevated risk of neonatal sepsis and severe infections until the age of 12 years, particularly in the extremely preterm group.^11^^,^^24^ A Finnish population study reported twice the risk of hospital admissions due to lower RTI in children up to 7 years of age who were born preterm at weeks 32–36 than those who were born at full-term.^25^ An Australian registry linkage study with follow-up from birth to 18 years of age found that gestational age was inversely related to infection-related hospitalisations through adolescence, with an overall estimated 12% increase in risk per week of gestation.^12^ A Danish population follow-up of 1 million individuals supported previous findings that preterm born had higher risk of hospital admissions for RTI that decreased with age, but found that the association ultimately disappeared during adolescence.^13^

While previous studies have focused on infections in children and adolescents, this study determined the risk of infections from birth until the age of 50 years. A Swedish population-based study of 674,820 persons born between 1973 and 1979 who were followed for up 36 years found that preterm birth was associated with increased mortality attributed to infections after 6 years of age but lacked power to conclude about risk among adults.^26^^,^^27^ To the best of our knowledge, the present study is the first to robustly examine the association between different gestational age groups at birth and the risk of hospitalisations for infectious diseases, both overall and for groups of infections, at ages from childhood up to adulthood. The underlying causes of this seemingly long-term and overall infection risk associated with birth before term remain largely unknown.

The immature immune system of preterm infants may increase their susceptibility to infection. The immune system undergoes significant maturation in the last trimester of pregnancy. Hence, preterm infants do not have the same level of immune competence and defence against microbes as full-term infants. Moreover, preterm infants may be exposed to several interventions such as caesarean section, prenatal and neonatal antibiotics and corticosteroids, intravenous lines, and invasive ventilatory support, potentially affecting their microbial composition, shaping of the immune system and organ development. Programming of the immune system is further supported by observations of lower rates of atopy in adults born preterm and at very low birthweight.^28^ How preterm birth affects the developing immune system and response to infections is an active area of research.^29^, ^30^, ^31^, ^32^

The link between preterm birth and long-term risk of respiratory infections, particularly lung infections, seems plausible because preterm birth is strongly associated with lung immaturity and the need of ventilatory support in the neonatal period.^33^ For preterm born, lower lung volumes and higher rates of chronic lung disease have been reported through childhood and into adulthood,^33^, ^34^, ^35^, ^36^, ^37^, ^38^ and associations between preterm birth and RTI risk in childhood and adolescence have been described previously.^25^^,^^28^^,^^34^^,^^36^ Reduced respiratory organ function and chronic lung disease increase the risk of RTIs in children as well as in adults, and an increased risk of chronic lung disease (CLD) in childhood and adulthood may, at least in part, mediate the excess RTI risk in children and adults born preterm.^38^ However, the increased risk of RTI and CLD in children born preterm are likely intertangled and cannot be solely explained by structural changes in the lung. Although the precise mechanisms are not yet fully understood, it is likely that the development of lung disease in early life is also connected to the establishment of the microbiota and the maturation of the immune system.^39^ Our findings underscores the need to evaluate risk in other populations and to study the efficacy of potential preventive measures, such as respiratory syncytial virus (RSV) vaccines and extended half-life monoclonal antibodies (e.g., nirsevimab).^40^

Understanding the link between shorter length of gestation and increased risk of severe infections can help tailor preventive and therapeutic measures, such as vaccinations, diagnostics, and medical interventions. This knowledge is particularly valuable during epidemics and pandemics when the identification of risk groups and prioritization of resources is necessary.

Advancements in prenatal and neonatal care have improved the survival rates in preterm births. In high-income countries, this development has been particularly related to improvements in the treatment of neonatal lung disease with surfactants since the 1990s.^33^ An analysis of nation-wide neonatal care in Norway reported that improved neonatal survival from 1967 to 2011 was mainly attributed to improved respiratory practices.^41^ Thus, ventilators for newborns were introduced in the 1970s, antenatal steroids in the 1980s and by the early 1990s, surfactant treatment was available at all neonatal departments. This suggests that the majority of individuals in the 30–50 year age group included in our study had access to neonatal units that provided treatment for neonatal lung disease. However, lung protective strategies such as surfactant therapy and continuous positive airway pressure (CPAP) were in the present study primarily applicable to individuals that were younger than 30 years of age at follow-up. The rarity of extremely preterm births and recency of improved survival for this group make it challenging to robustly assess their adult disease risk, as results may be impacted by survivor bias, leading to estimates for the most extreme preterm born several decades ago, biased towards the null. Thus in the present analysis, follow-up time started in 2008 for all, consequently the association between prematurity and infection risk for the oldest cohorts might be influenced by the death of the frailest individuals before the measurement of person-time begins. If this scenario occurs, it will lead to a weakened association. Consequently, the associations identified in our analyses would reflect conservative estimates in the older age groups.

From a global and population perspective, survival rates have improved, particularly for those born in the moderate to late term.^33^^,^^42^^,^^43^ Therefore, the largest number of preterm survivors belong to older gestational age groups, many of whom also survived earlier, and the present findings are of high public health relevance. However, it is possible that we will see fewer adverse outcomes especially in the lower gestational age groups through improved care practices in a potentially optimistic scenario.

Misclassification of gestational age is a potential limitation although we applied inclusion criteria based on possible SD scores for birth weight related to specific weeks of gestation to reduce this risk. Further, different methods of estimating gestational age may influence the accuracy of the estimates of gestational age.^44^ Compared with estimates of gestational age based on last menstrual period it has been found that estimates based on ultrasound shifted the entire distribution of gestational age to lower gestational ages.^45^ Consequently, this could have produced less contrast and more conservative estimates, particularly for individuals born in the earlier years of the study period when ultrasound was not used routinely.

Another limitation is the relatively low proportion of infections identified as being caused by specific microbes. We applied a strict definition where a specific pathogen or a typical clinical syndrome (e.g., bronchiolitis) had to be specified by the specialist care doctor at discharge for an infection to be classified as bacterial or viral. In clinical practice, the specific pathogen can often not be identified. In patients hospitalized with pneumonia, a pathogen could be identified in 40–60% of cases even with comprehensive microbiological testing,^46^^,^^47^ an indication that our data defining etiologies for around 30% of RTI admissions is in line with clinical practice using modern microbiological testing. The extent to which diagnoses are organ- or pathogen-specific varies according to age and other factors. For example, bronchiolitis caused by RSV is the dominant cause of hospitalisation in infants and often results in virus-specific diagnoses. This may have improved the accuracy of the association between preterm birth and viral infections in infancy. The lack of comparably strong associations between older children and adults likely reflects both a true reduction in risk with age and a loss of precision due to fewer virus-specific diagnoses. Overall, limited pathogen- and organ-specific diagnoses impede the hypothesis testing of specific endpoints. As a result, the findings have been reported in broader categories, such as RTIs versus non-RTIs and viral versus bacterial infections. Finally, possible residual confounders and mediators that could explain long-term infectious disease susceptibility in preterm births were not considered. Further studies should evaluate the role of factors such as perinatal infections, social conditions, and lifestyle and genetic factors. The role of underlying chronic diseases on the risk of infection in this group needs to be assessed. Nevertheless, due to large sampling requirements and considerable lead-time to the potential outcome, infection-related hospitalisations among individuals ≤50 years, record-linkage analysis is an appropriate methodology to identify associations for perinatal factors and adult disease. Despite the advantages of the large dataset providing high precision, potential unknown dependencies in the data may still introduce limitations. High-quality data from the MBRN enabled the assessment of associations between preterm birth and infectious diseases while adjusting for maternal factors, socioeconomic status, and other perinatal factors. Multiple decades of low perinatal mortality in Norway make the population uniquely suited for studying the long-term consequences of preterm birth.

In conclusion, children, adolescents, and adults have a gradually higher risk of infection-related hospitalisations with shorter gestational age at birth. The risk patterns were similar across infectious disease groups, such as bacterial and viral infections, and were not only evident for RTIs but also for infections not classified as RTI. The mechanisms involved are mainly understudied but may be linked to early life immune system and organ system development. Nevertheless, current knowledge may be used to emphasize preventive lifestyles, such as avoidance of smoking and obesity, optimization of hygiene habits, and further develop relevant risk stratified immunization recommendations.

## Contributors

KR is the guarantor of the study and affirms that the manuscript is an honest, accurate, and transparent account of the study being reported; that no important aspects of the study have been omitted. All authors participated in critically reviewing the analyses and the report and interpreting the findings. JV and SMN analyzed the data, interpreted the data, drafted the initial manuscript, and revised the manuscript. KR, SMN, SS, ES, TR, AA designed the study and statistical methods and analyses and reviewed and revised the analyses and manuscript for important intellectual content. HD, SO, JKD, EK, JM planned the study, and reviewed and revised the analysis and manuscript for important intellectual content. KR is responsible for the Norwegian data, designed and planned the study design, drafted the initial manuscript, revised and approved the final manuscript. KR and ES were resposible for conceptualization and funding. KR, JV and SMN had full access to all the data in the study, verified the data, and takes responsibility for the integrity of the data and the accuracy of the data analysis. All authors take final responsibility for the decision to submit the report for publication.

## Data sharing statement

Data protection law precludes us from sharing the data used in this study. Access to the data can be requested from the Norwegian Health Data service (www.helsedata.no).

## Declaration of interests

All authors declare no competing interests.

## References

[1] Kwok A.J., Mentzer A., Knight J.C. (2021). Host genetics and infectious disease: new tools, insights and translational opportunities. Nat Rev Genet.

[2] Ponsford M.J., Gkatzionis A., Walker V.M. (2020). Cardiometabolic traits, sepsis, and severe COVID-19: a Mendelian randomization investigation. Circulation.

[3] Risnes K., Bilsteen J.F., Brown P. (2021). Mortality among young adults born preterm and early term in 4 Nordic nations. JAMA Netw Open.

[4] Chawanpaiboon S., Vogel J.P., Moller A.B. (2019). Global, regional, and national estimates of levels of preterm birth in 2014: a systematic review and modelling analysis. Lancet Glob Health.

[5] Kajantie E., Strang-Karlsson S., Evensen K.A.I., Haaramo P. (2019). Adult outcomes of being born late preterm or early term - what do we know?. Semin Fetal Neonatal Med.

[6] Liu L., Johnson H.L., Cousens S. (2012). Global, regional, and national causes of child mortality: an updated systematic analysis for 2010 with time trends since 2000. Lancet.

[7] Stensvold H.J., Klingenberg C., Stoen R. (2017). Neonatal morbidity and 1-year survival of extremely preterm infants. Pediatrics.

[8] Hovi P., Andersson S., Eriksson J.G. (2007). Glucose regulation in young adults with very low birth weight. N Engl J Med.

[9] Moster D., Lie R.T., Markestad T. (2008). Long-term medical and social consequences of preterm birth. N Engl J Med.

[10] Crump C., Howell E.A., Stroustrup A., McLaughlin M.A., Sundquist J., Sundquist K. (2019). Association of preterm birth with risk of ischemic heart disease in adulthood. JAMA Pediatr.

[11] Coathup V., Boyle E., Carson C. (2020). Gestational age and hospital admissions during childhood: population based, record linkage study in England (TIGAR study). BMJ.

[12] Miller J.E., Hammond G.C., Strunk T. (2016). Association of gestational age and growth measures at birth with infection-related admissions to hospital throughout childhood: a population-based, data-linkage study from Western Australia. Lancet Infect Dis.

[13] Garioud A.L.B., Skoven F.H., Gregersen R., Lange T., Buchvald F., Greisen G. (2020). The increased susceptibility to airway infections after preterm birth does not persist into adolescence. PLoS One.

[14] Medical birth registry of Norway (2022). Norwegian Institute of Public Health. https://www.fhi.no/en/hn/health-registries/medical-birth-registry-of-norway/.

[15] Laugesen K., Ludvigsson J.F., Schmidt M. (2021). Nordic health registry-based research: a review of health care systems and key registries. Clin Epidemiol.

[16] Bakken I.J., Ariansen A.M.S., Knudsen G.P., Johansen K.I., Vollset S.E. (2020). The Norwegian patient registry and the Norwegian registry for primary health care: research potential of two nationwide health-care registries. Scand J Public Health.

[17] (2022). Norwegian cause of death registry. Norwegian Institute of Public Health. https://www.fhi.no/en/hn/health-registries/cause-of-death-registry/.

[18] (2022). Statistics Norway. SSB. https://www.ssb.no/en.

[19] Pedersen A.G., Ellingsen C.L. (2015). Data quality in the causes of death registry. Tidsskr Nor Laegeforen.

[20] Marsál K., Persson P.H., Larsen T., Lilja H., Selbing A., Sultan B. (1996). Intrauterine growth curves based on ultrasonically estimated foetal weights. Acta Paediatr.

[21] Pawitan Y. (2001).

[22] StataCorp (2021).

[23] Hunter J.D. (2007). Matplotlib: a 2D graphics environment. Comput Sci Eng.

[24] Giannoni E., Agyeman P.K.A., Stocker M. (2018). Neonatal sepsis of early onset, and hospital-acquired and community-acquired late onset: a prospective population-based cohort study. J Pediatr.

[25] Haataja P., Korhonen P., Ojala R. (2018). Hospital admissions for lower respiratory tract infections in children born moderately/late preterm. Pediatr Pulmonol.

[26] Crump C., Sundquist K., Sundquist J. (2012). Prematurity and mortality in childhood and early adulthood—reply. JAMA.

[27] Crump C. (2020). An overview of adult health outcomes after preterm birth. Early Hum Dev.

[28] Siltanen M., Wehkalampi K., Hovi P. (2011). Preterm birth reduces the incidence of atopy in adulthood. J Allergy Clin Immunol.

[29] Marchant E.A., Kan B., Sharma A.A. (2015). Attenuated innate immune defenses in very premature neonates during the neonatal period. Pediatr Res.

[30] de Jong E., Strunk T., Burgner D., Lavoie P.M., Currie A. (2017). The phenotype and function of preterm infant monocytes: implications for susceptibility to infection. J Leukoc Biol.

[31] Dietz S., Molnar K., Riedel H. (2023). Expression of immune checkpoint molecules on adult and neonatal T-cells. Immunol Res.

[32] Henneke P., Kierdorf K., Hall L.J., Sperandio M., Hornef M. (2021). Perinatal development of innate immune topology. Elife.

[33] Owen L.S., Manley B.J., Davis P.G., Doyle L.W. (2017). The evolution of modern respiratory care for preterm infants. Lancet.

[34] Bårdsen T., Røksund O.D., Benestad M.R. (2022). Tracking of lung function from 10 to 35 years after being born extremely preterm or with extremely low birth weight. Thorax.

[35] Satrell E., Clemm H., Røksund O.D. (2022). Development of lung diffusion to adulthood following extremely preterm birth. Eur Respir J.

[36] Doyle L.W., Andersson S., Bush A. (2019). Expiratory airflow in late adolescence and early adulthood in individuals born very preterm or with very low birthweight compared with controls born at term or with normal birthweight: a meta-analysis of individual participant data. Lancet Respir Med.

[37] Backman H., Bhatta L., Hedman L. (2019). Asthma is still a risk factor for mortality in Sweden and Norway – the Nordic EpiLung Study. Eur Respir J.

[38] Pulakka A., Risnes K., Metsälä J. (2023). Preterm birth and asthma and COPD in adulthood: a nationwide register study from two Nordic countries. Eur Respir J.

[39] Griffin M.P., Yuan Y., Takas T. (2020). Single-dose nirsevimab for prevention of RSV in preterm infants. N Engl J Med.

[40] Anderson J., Do L.A.H., Wurzel D., Licciardi P.V. (2023). Understanding the increased susceptibility to asthma development in preterm infants. Allergy.

[41] Grytten J., Monkerud L., Skau I., Eskild A., Sørensen R.J., Saugstad O.D. (2017). Saving newborn babies – the benefits of interventions in neonatal care in Norway over more than 40 years. Health Econ.

[42] Hug L., Alexander M., You D., Alkema L., UN (2019). National, regional, and global levels and trends in neonatal mortality between 1990 and 2017, with scenario-based projections to 2030: a systematic analysis. Lancet Glob Health.

[43] Stoll B.J., Hansen N.I., Bell E.F. (2015). Trends in care practices, morbidity, and mortality of extremely preterm neonates, 1993–2012. JAMA.

[44] Moth F.N., Sebastian T.R., Horn J., Rich-Edwards J., Romundstad P.R., Åsvold B.O. (2016). Validity of a selection of pregnancy complications in the medical birth registry of Norway. Acta Obstet Gynecol Scand.

[45] Yang H., Kramer M.S., Platt R.W. (2002). How does early ultrasound scan estimation of gestational age lead to higher rates of preterm birth?. Am J Obstet Gynecol.

[46] Templeton K.E., Scheltinga S.A., van den Eeden W.C., Graffelman W.A., van den Broek P.J., Claas E.C.J. (2005). Improved diagnosis of the etiology of community-acquired pneumonia with real-time polymerase chain reaction. Clin Infect Dis.

[47] Johansson N., Kalin M., Tiveljung-Lindell A., Giske C.G., Hedlund J. (2010). Etiology of community-acquired pneumonia: increased microbiological yield with new diagnostic methods. Clin Infect Dis.

