# Supplementary material for: Gestational age at birth and hospitalisations for infections among individuals aged 0–50 years in Norway: a longitudinal, register-based, cohort study
Source: eClinicalMedicine. 2023 Jul 20;62:102108. doi: 10.1016/j.eclinm.2023.102108 (PMC10393616; doi:10.1016/j.eclinm.2023.102108)
Supplement: Supplementary material [file mmc1.docx]

**Part I: data material**

Supplemental table 1. ICD-10 codes for defining outcomes.

Supplemental table 2. Hospitalisation rate ratio for hospitalisations due to any infectious disease among males and females born at different gestational age (in weeks), and p for difference.

Supplemental table 3. The number of hospitalisations and persons hospitalised due to any infectious diseases, and person-years at risk of hospitalisation.

Supplemental table 4. Yearly rates for hospitalisations per 1,000 person-years (2008-2017).

Supplemental table 5. The number of hospitalisations and persons hospitalised due to respiratory tract infections, and person-years at risk of admission.

Supplemental table 6. The number of hospitalisations and persons hospitalised due to viral infectious diseases, and person-years at risk of hospitalisation.

Supplemental table 7. The number of hospitalisations and persons hospitalised due to bacterial infectious diseases, and person-years at risk of hospitalisation.

Supplemental figure 1. Age distribution and birth years during the follow up period.

**Part II: supplemental outcomes**

Supplemental figure 2.  Estimated yearly rates and rate ratios for hospitalisations related to viral respiratory tract infections (2008-2017).

Supplemental figure 3. Estimated yearly rates (per 1,000 person-years) and rate ratios for hospitalisations related to bacterial respiratory tract infections (2008-2017)

Supplemental figure 4. Estimated yearly rates (per 1,000 person-years) and rate ratios for hospitalisations related to any infectious diseases, excluding respiratory tract infections (2008-2017).

Supplemental figure 5. Estimated yearly rates (per 1,000 person-years) and rate ratios for hospitalisations related to viral infectious diseases, excluding respiratory tract infections (2008-2017).

Supplemental figure 6. Estimated yearly rates (per 1,000 person-years) and rate ratios for hospitalisations related to bacterial infectious diseases, excluding respiratory tract infections (2008-2017).

**Part III: additional analyses on any infections**

Supplemental figure 7. Estimated yearly rates (per 1,000 person-years) and rate ratios for hospitalisations related to any infectious diseases, adjusted for birth weight SD-score (2008-2017).

Supplemental figure 8. Estimated yearly rates (per 1,000 person-years) and rate ratios for hospitalisations related to any infectious diseases, adjusted for maternal education (2008-2017).

Supplemental figure 9. Estimated yearly rates (per 1,000 person-years) and rate ratios for hospitalisations related to any infectious diseases, adjusted for maternal country of birth (2008-2017).

Supplemental figure 10. Estimated yearly rates (per 1,000 person-years) and rate ratios for hospitalisations related to any infectious diseases, adjusted for birth month (2008-2017).

Supplemental figure 11. Estimated yearly rates (per 1,000 person-years) and rate ratios for hospitalisations related to any infectious diseases, excluding congenital malformations (2008-2017).

Supplemental figure 12. Estimated yearly rates (per 1,000 person-years) and rate ratios for hospitalisations related to any infectious diseases, excluding any CP/severe disability (defined at any time in the Norwegian patient registry) (2008-2017).

Supplemental figure 13. Estimated yearly rates (per 1,000 person-years) and rate ratios for hospitalisations related to any infectious diseases, excluding multiples (2008-2017).

**Part IV: overview**

Supplemental figure 14. Estimated hospitalisation rates any infectious diseases, respiratory tract infections and any infectious diseases excluding respiratory tract diseases (2008-2017).

**Part I: data material**

Supplemental table 1. ICD-10 codes for defining outcomes.

| Any infectious disease |
| --- |
| A;B;G00;G01;G02;G03;G04;G05;G06;G07;G08;H050;H600;H602;H603;H609;H610;H700;I00;I01;I301;I330;I339;I38;I39;I400;I400;I80;I408;J00;J01;J02;J03;J04;J05;J06;J09;J10;J11;J12;J13;J14;J15;J16;J17;J18;J20;J21;J22;J32;J390;J391;J85;J86;K040;K044;K046;K047;K050;K052;K112;K113;K122;K35;K36;K37;K65;K750;K751;K800;K803;K804;K81;K830;L0;M00;M01;M02;M03;M600;M726;M86;N10;N11;N12;N300;N302;N34;N390;N410;N450;N459;N61;N70;N71;N729;N730;N732;N733;N738;N74;N751;N760;N762;N764;N768;N980;O080;O230;O231;O233;O234;O411;O752;O753;O85;O86;O883;O91;P35;P36;P37;P38;P39;R572;R650;R65;R651;T814;T802;T826;T827;T835;T845;T846;T847;T857;T880;U04;U04 |
| Viral infectious diseases |
| J09;J10;J11;J12;U04;A8;A92;A93;A94;A95;A96;A97;A98;A99;B0;B15;B16;B17;B18;B19;B20;B21;B22;B23;B24;B25;B26;B27;B28;B29;B30;B31;B32;B33;B34;B97;J171;J203;J204;J205;J206;J207;J210;J211 |
| Bacterial infectious diseases |
| J13;J14;J150;J151;J152;J153;J154;J155;J156;J157;J158;J159;J16;J170;J200;J201;J202;A481;K350;K37;K650;K800;K803;K804;K810;K830;N10;N12;N390;A39;G000;G001;G002;G003;G008;G009;G01;G042;A46;L031;L032;L033;L038;L035;M726;M00;M013;M860;M861;M862;M869;A40;A41;A483;R572;B95;B96;I330;I339;I38 |
| Respiratory tract infections |
| J0;J1;J20;J21;J22;J32;J390;J391;J85;J86 |
| Any infectious disease, excluding respiratory tract infections |
| A;B;G00;G01;G02;G03;G04;G05;G06;G07;G08;H050;H600;H602;H603;H609;H610;H700;I00;I01;I301;I330;I339;I38;I39;I400;I400;I80;I408;K040;K044;K046;K047;K050;K052;K112;K113;K122;K35;K36;K37;K65;K750;K751;K800;K803;K804;K81;K830;L0;M00;M01;M02;M03;M600;M726;M86;N10;N11;N12;N300;N302;N34;N390;N410;N450;N459;N61;N70;N71;N729;N730;N732;N733;N738;N74;N751;N760;N762;N764;N768;N980;O080;O230;O231;O233;O234;O411;O752;O753;O85;O86;O883;O91;P35;P36;P37;P38;P39;R572;R650;R65;R651;T814;T802;T826;T827;T835;T845;T846;T847;T857;T880;U04;U04 |
| Viral infectious diseases, excluding respiratory tract infections |
| U04;A8;A92;A93;A94;A95;A96;A97;A98;A99;B0;B15;B16;B17;B18;B19;B20;B21;B22;B23;B24;B25;B26;B27;B28;B29;B30;B31;B32;B33;B34;B97 |
| Bacterial infectious diseases, excluding respiratory tract infections |
| A481;K350;K37;K650;K800;K803;K804;K810;K830;N10;N12;N390;A39;G000;G001;G002;G003;G008;G009;G01;G042;A46;L031;L032;L033;L038;L035;M726;M00;M013;M860;M861;M862;M869;A40;A41;A483;R572;B95;B96;I330;I339;I38;A40;A41;A483;A021;A207;A217;A227;A241;A267;A282;A327;A394;A427;A5486;R572;R651;R650;O753;O85 |
| Bacterial respiratory tract infections |
| J13;J14;J150;J151;J152;J153;J154;J155;J156;J157;J158;J159;J160;J170;J200;J201;J202 |
| Viral respiratory tract infections |
| J09;J10;J11;J12;U04;J203;J204;J205;J206;J207;J210;J211;J171 |

**Part I: data material**

Supplemental table 2. Hospitalisation rate ratio for hospitalisations due to any infectious disease among males and females born at different gestational age (in weeks), and p for difference.

|  | Males |  | Females |  | *p for diff.* |
| --- | --- | --- | --- | --- | --- |
| 0 to 11 months |  |  |  |  |  |
| Extremely preterm (23-27) | 5,4 |  | 6,7 |  | *0,11* |
| Very preterm (28-31) | 3,6 |  | 4,2 |  | *0,07* |
| Moderately preterm (32-33) | 2,6 |  | 2,9 |  | *0,18* |
| Late preterm (34-36) | 1,7 |  | 1,8 |  | *0,47* |
| Early term (37-38) | 1,3 |  | 1,3 |  | *0,79* |
| Full term (39-41) | *Ref.* |  | *Ref.* |  | *Ref.* |
| Post term (42- 44) | 0,9 |  | 1,0 |  | *0,56* |
| 1 to 5 years |  |  |  |  |  |
| Extremely preterm (23-27) | 7,6 |  | 7,3 |  | *0,74* |
| Very preterm (28-31) | 3,1 |  | 3,7 |  | *0,06* |
| Moderately preterm (32-33) | 2,0 |  | 2,5 |  | *0,04* |
| Late preterm (34-36) | 1,6 |  | 1,7 |  | *0,62* |
| Early term (37-38) | 1,4 |  | 1,2 |  | *0,00* |
| Full term (39-41) | *Ref.* |  | *Ref.* |  | *Ref.* |
| Post term (42- 44) | 1,0 |  | 0,9 |  | *0,43* |
| 6 to 14 years |  |  |  |  |  |
| Extremely preterm (23-27) | 3,5 |  | 2,0 |  | *0,03* |
| Very preterm (28-31) | 1,7 |  | 1,7 |  | *0,90* |
| Moderately preterm (32-33) | 1,3 |  | 1,3 |  | *0,86* |
| Late preterm (34-36) | 1,3 |  | 1,2 |  | *0,40* |
| Early term (37-38) | 1,2 |  | 1,1 |  | *0,07* |
| Full term (39-41) | *Ref.* |  | *Ref.* |  | *Ref.* |
| Post term (42- 44) | 1,1 |  | 1,0 |  | *0,12* |
| 15 to 29 years |  |  |  |  |  |
| Extremely preterm (23-27) | 2,3 |  | 1,5 |  | *0,26* |
| Very preterm (28-31) | 1,4 |  | 1,3 |  | *0,82* |
| Moderately preterm (32-33) | 1,1 |  | 1,2 |  | *0,53* |
| Late preterm (34-36) | 1,1 |  | 1,1 |  | *0,98* |
| Early term (37-38) | 1,0 |  | 1,1 |  | *0,39* |
| Full term (39-41) | *Ref.* |  | *Ref.* |  | *Ref.* |
| Post term (42- 44) | 1,0 |  | 1,1 |  | *0,02* |
| 30 to 50 years |  |  |  |  |  |
| Extremely preterm (23-27) | 1,5 |  | 0,8 |  | *0,20* |
| Very preterm (28-31) | 1,5 |  | 1,4 |  | *0,67* |
| Moderately preterm (32-33) | 1,3 |  | 1,3 |  | *1,00* |
| Late preterm (34-36) | 1,2 |  | 1,1 |  | *0,30* |
| Early term (37-38) | 1,1 |  | 1,1 |  | *0,85* |
| Full term (39-41) | *Ref.* |  | *Ref.* |  | *Ref.* |
| Post term (42- 44) | 1,0 |  | 1,1 |  | *0,38* |

Adjusted for maternal age, parity, and year of birth.

**Part I: data material**

Supplemental table 3. The number of hospitalisations and persons hospitalised due to any infectious diseases, and person-years at risk of hospitalisation.

|  | **Gestational age at birth in weeks** | | | | | | | |
| --- | --- | --- | --- | --- | --- | --- | --- | --- |
|  | **23 to 27** | **28 to 31** | **32 to 33** | **34 to 36** | **37 to 38** | **39 to 41** | **42 to 44** | **Total** |
| **0 to 11 months** | |  |  |  |  |  |  |  |
| Hospitalisations | 479 | 877 | 850 | 3 165 | 9 339 | 29 154 | 1 754 | 45 618 |
| Persons | 298 | 592 | 583 | 2 335 | 7 219 | 23 691 | 1 436 | 36 154 |
| Person-years | 1 238 | 3 513 | 4 682 | 25 472 | 101 293 | 404 388 | 26 933 | 567 519 |
| **1 to 5 years** |  |  |  |  |  |  |  |  |
| Hospitalisations | 875 | 1 129 | 1 048 | 4 124 | 12 533 | 38 165 | 2 914 | 60 788 |
| Persons | 398 | 707 | 711 | 2 886 | 9 191 | 29 690 | 2 199 | 45 782 |
| Person-years | 6 301 | 18 758 | 25 673 | 134 919 | 524 140 | 2 043 574 | 165 447 | 2 918 812 |
| **6 to 14 years** |  |  |  |  |  |  |  |  |
| Hospitalisations | 162 | 284 | 307 | 1 557 | 4 999 | 17 722 | 2 306 | 27 337 |
| Persons | 87 | 198 | 233 | 1 124 | 3 773 | 13 873 | 1 810 | 21 098 |
| Person-years | 10 972 | 33 854 | 45 358 | 234 273 | 855 632 | 3 350 127 | 414 314 | 4 944 530 |
| **15 to 29 years** | |  |  |  |  |  |  |  |
| Hospitalisations | 168 | 534 | 688 | 3 598 | 11 458 | 55 529 | 11 189 | 83 164 |
| Persons | 85 | 347 | 475 | 2 567 | 8 453 | 41 910 | 8 389 | 62 226 |
| Person-years | 9 498 | 38 632 | 55 988 | 308 154 | 1 026 272 | 5 050 416 | 981 804 | 7 470 764 |
| **30 to 50 years** | |  |  |  |  |  |  |  |
| Hospitalisations | 34 | 451 | 696 | 3 960 | 12 007 | 65 490 | 14 395 | 97 033 |
| Persons | 22 | 261 | 411 | 2 465 | 7 701 | 43 493 | 9 341 | 63 694 |
| Person-years | 2 677 | 27 436 | 47 722 | 293 865 | 989 198 | 5 705 140 | 1 175 306 | 8 241 344 |

**Part I: data material**

Supplemental table 4. Yearly rates for hospitalisations per 1,000 person-years (2008-2017).

|  |  | 0-11 months | 1-5 years | 6-14 years | 15-29 years | 30-50 years |
| --- | --- | --- | --- | --- | --- | --- |
|  | Person-years | 567 519 | 2 918 812 | 4 944 530 | 7 470 764 | 8 241 344 |
| Any infectious disease | Hospitalisations | 45 618 | 60 788 | 27 337 | 83 164 | 97 033 |
|  | Rate | 80·4 | 20·8 | 5·5 | 11·1 | 11·8 |
| Respiratory tract infections (RTI) | Hospitalisations | 28 038 | 34 114 | 6 809 | 16 945 | 21 474 |
|  | Rate | 49·4 | 11·7 | 1·4 | 2·3 | 2·6 |
| Viral infectious diseases | Hospitalisations | 12 939 | 10 501 | 2 620 | 8 678 | 6 329 |
|  | Rate | 22·8 | 3·6 | 0·5 | 1·2 | 0·8 |
| Bacterial infectious diseases | Hospitalisations | 5 346 | 10 735 | 6 613 | 19 927 | 33 427 |
|  | Rate | 9·4 | 3·7 | 1·3 | 2·7 | 4·1 |
| Viral RTIs | Hospitalisations | 10 692 | 7 517 | 878 | 1 449 | 1 758 |
|  | Rate | 18·8 | 2·6 | 0·2 | 0·2 | 0·2 |
| Bacterial RTIs | Hospitalisations | 718 | 4 466 | 1 715 | 5 134 | 9 147 |
|  | Rate | 1·3 | 1·5 | 0·3 | 0·7 | 1·1 |
| Any infections not RTIs | Hospitalisations | 17 590 | 26 686 | 20 537 | 66 246 | 75 624 |
|  | Rate | 31·0 | 9·1 | 4·2 | 8·9 | 9·2 |
| Viral infections not RTIs | Hospitalisations | 2 247 | 2 984 | 1 742 | 7 230 | 4 571 |
|  | Rate | 4·0 | 1·0 | 0·4 | 1·0 | 0·6 |
| Bacterial infections not RTIs | Hospitalisations | 4 628 | 6 264 | 4 891 | 15 639 | 25 215 |
|  | Rate | 8·2 | 2·1 | 1·0 | 2·1 | 3·1 |

**Part I: data material**

Supplemental table 5. The number of hospitalisations and persons hospitalised due to respiratory tract infections, and person-years at risk of hospitalisations.

|  | **Gestational age at birth in weeks** | | | | | | | |
| --- | --- | --- | --- | --- | --- | --- | --- | --- |
|  | **23 to 27** | **28 to 31** | **32 to 33** | **34 to 36** | **37 to 38** | **39 to 41** | **42 to 44** | **Total** |
| **0 to 11 months** |  |  |  |  |  |  |  |  |
| Hospitalisations | 384 | 672 | 642 | 2 321 | 6 174 | 16 930 | 915 | 28 038 |
| Persons | 247 | 474 | 451 | 1 749 | 4 949 | 14 123 | 764 | 22 757 |
| Person-years | 1 238 | 3 513 | 4 682 | 25 472 | 101 293 | 404 388 | 26 933 | 567 519 |
| **1 to 5 years** |  |  |  |  |  |  |  |  |
| Hospitalisations | 742 | 821 | 708 | 2 463 | 7 148 | 20 643 | 1 589 | 34 114 |
| Persons | 341 | 521 | 494 | 1 796 | 5 502 | 16 849 | 1 239 | 26 742 |
| Person-years | 6 301 | 18 758 | 25 673 | 134 919 | 524 140 | 2 043 574 | 165 447 | 2 918 812 |
| **6 to 14 years** |  |  |  |  |  |  |  |  |
| Hospitalisations | 74 | 111 | 94 | 501 | 1 419 | 4 065 | 545 | 6 809 |
| Persons | 44 | 71 | 70 | 343 | 1 049 | 3 191 | 407 | 5 175 |
| Person-years | 10 972 | 33 854 | 45 358 | 234 273 | 855 632 | 3 350 127 | 414 314 | 4 944 530 |
| **15 to 29 years** |  |  |  |  |  |  |  |  |
| Hospitalisations | 51 | 128 | 167 | 817 | 2 420 | 11 092 | 2 270 | 16 945 |
| Persons | 21 | 83 | 116 | 596 | 1 842 | 8 718 | 1 772 | 13 148 |
| Person-years | 9 498 | 38 632 | 55 988 | 308 154 | 1 026 272 | 5 050 416 | 981 804 | 7 470 764 |
| **30 to 50 years** |  |  |  |  |  |  |  |  |
| Hospitalisations | 12 | 105 | 152 | 929 | 2 682 | 14 508 | 3 086 | 21 474 |
| Persons | 7 | 71 | 107 | 664 | 1 943 | 10 862 | 2 315 | 15 969 |
| Person-years | 2 677 | 27 436 | 47 722 | 293 865 | 989 198 | 5 705 140 | 1 175 306 | 8 241 344 |

**Part I: data material**

Supplemental table 6. The number of hospitalisations and persons hospitalised due to viral infectious diseases, and person-years at risk of hospitalisations.

|  | **Gestational age at birth in weeks** | | | | | | | |
| --- | --- | --- | --- | --- | --- | --- | --- | --- |
|  | **23 to 27** | **28 to 31** | **32 to 33** | **34 to 36** | **37 to 38** | **39 to 41** | **42 to 44** | **Total** |
| **0 to 11 months** |  |  |  |  |  |  |  |  |
| Hospitalisations | 108 | 284 | 243 | 1 075 | 2 836 | 7 982 | 411 | 12 939 |
| Persons | 90 | 231 | 202 | 905 | 2 467 | 7 084 | 359 | 11 338 |
| Person-years | 1 238 | 3 513 | 4 682 | 25 472 | 101 293 | 404 388 | 26 933 | 567 519 |
| **1 to 5 years** |  |  |  |  |  |  |  |  |
| Hospitalisations | 293 | 328 | 229 | 756 | 2 135 | 6 307 | 453 | 10 501 |
| Persons | 182 | 238 | 182 | 628 | 1 814 | 5 514 | 387 | 8 945 |
| Person-years | 6 301 | 18 758 | 25 673 | 134 919 | 524 140 | 2 043 574 | 165 447 | 2 918 812 |
| **6 to 14 years** |  |  |  |  |  |  |  |  |
| Hospitalisations | 31 | 33 | 27 | 139 | 452 | 1 720 | 218 | 2 620 |
| Persons | 22 | 26 | 22 | 124 | 392 | 1 489 | 196 | 2 271 |
| Person-years | 10 972 | 33 854 | 45 358 | 234 273 | 855 632 | 3 350 127 | 414 314 | 4 944 530 |
| **15 to 29 years** |  |  |  |  |  |  |  |  |
| Hospitalisations | 14 | 39 | 64 | 392 | 1 221 | 5 804 | 1 144 | 8 678 |
| Persons | 13 | 35 | 60 | 341 | 1 067 | 5 119 | 993 | 7 628 |
| Person-years | 9 498 | 38 632 | 55 988 | 308 154 | 1 026 272 | 5 050 416 | 981 804 | 7 470 764 |
| **30 to 50 years** |  |  |  |  |  |  |  |  |
| Hospitalisations | 5 | 36 | 46 | 249 | 784 | 4 252 | 957 | 6 329 |
| Persons | 3 | 30 | 42 | 217 | 663 | 3 619 | 796 | 5 370 |
| Person-years | 2 677 | 27 436 | 47 722 | 293 865 | 989 198 | 5 705 140 | 1 175 306 | 8 241 344 |

**Part I: data material**

Supplemental table 7. The number of hospitalisations and persons hospitalised due to bacterial infectious diseases, and person-years at risk of hospitalisations.

|  | **Gestational age at birth in weeks** | | | | | | | |
| --- | --- | --- | --- | --- | --- | --- | --- | --- |
|  | **23 to 27** | **28 to 31** | **32 to 33** | **34 to 36** | **37 to 38** | **39 to 41** | **42 to 44** | **Total** |
| **0 to 11 months** | |  |  |  |  |  |  |  |
| Hospitalisations | 38 | 72 | 84 | 321 | 1 026 | 3 581 | 224 | 5 346 |
| Persons | 29 | 65 | 67 | 260 | 833 | 2 991 | 182 | 4 427 |
| Person-years | 1 238 | 3 513 | 4 682 | 25 472 | 101 293 | 404 388 | 26 933 | 567 519 |
| **1 to 5 years** |  |  |  |  |  |  |  |  |
| Hospitalisations | 83 | 146 | 172 | 672 | 2 241 | 6 872 | 549 | 10 735 |
| Persons | 68 | 114 | 125 | 483 | 1 689 | 5 300 | 415 | 8 194 |
| Person-years | 6 301 | 18 758 | 25 673 | 134 919 | 524 140 | 2 043 574 | 165 447 | 2 918 812 |
| **6 to 14 years** |  |  |  |  |  |  |  |  |
| Hospitalisations | 43 | 64 | 67 | 405 | 1 306 | 4 206 | 522 | 6 613 |
| Persons | 22 | 47 | 56 | 298 | 917 | 3 114 | 393 | 4 847 |
| Person-years | 10 972 | 33 854 | 45 358 | 234 273 | 855 632 | 3 350 127 | 414 314 | 4 944 530 |
| **15 to 29 years** | |  |  |  |  |  |  |  |
| Hospitalisations | 61 | 156 | 211 | 883 | 2 749 | 13 148 | 2 719 | 19 927 |
| Persons | 23 | 94 | 136 | 633 | 2 006 | 9 732 | 2 063 | 14 687 |
| Person-years | 9 498 | 38 632 | 55 988 | 308 154 | 1 026 272 | 5 050 416 | 981 804 | 7 470 764 |
| **30 to 50 years** | |  |  |  |  |  |  |  |
| Hospitalisations | 14 | 166 | 245 | 1 471 | 4 216 | 22 387 | 4 928 | 33 427 |
| Persons | 11 | 94 | 147 | 901 | 2 707 | 14 908 | 3 214 | 21 982 |
| Person-years | 2 677 | 27 436 | 47 722 | 293 865 | 989 198 | 5 705 140 | 1 175 306 | 8 241 344 |

**Part I: data material**


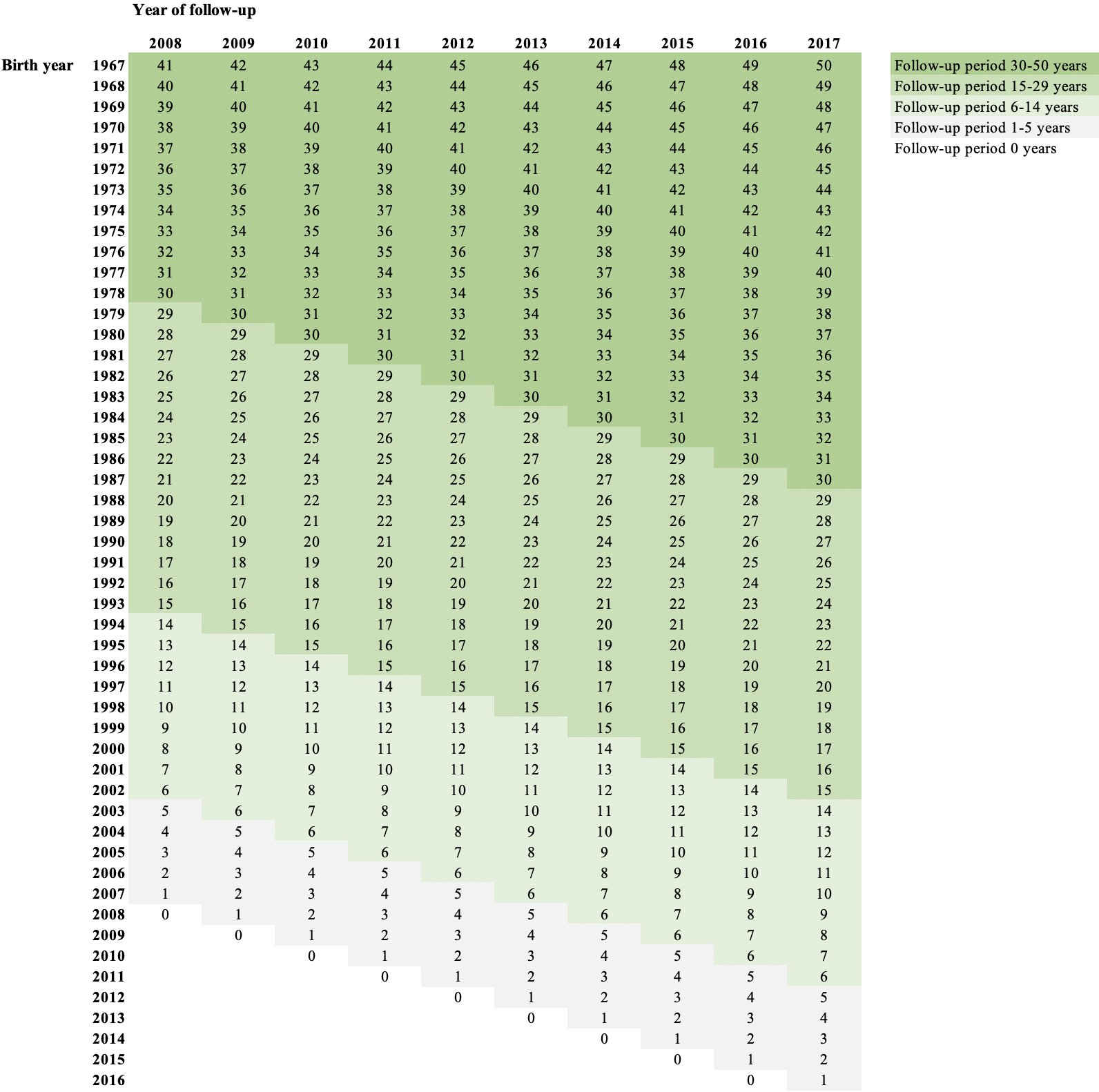


Supplemental figure 1. Age distribution and birth years during the follow-up period.

**Part II: supplemental outcomes**

Supplemental figure 2. Estimated yearly rates (per 1,000 person-years) and rate ratios for hospitalisations related to viral respiratory tract infections (2008-2017). Hospitalisation rate ratios (RRs) are plotted on a logarithmic scale and were calculated with individuals born at term (week 39–41) as references. The error bars represent the 95% confidence intervals (95% CI) for the RRs. The results are adjusted for year of birth, maternal age, parity, and sex.

**Part II: supplemental outcomes**

 

Supplemental figure 3. Estimated yearly rates (per 1,000 person-years) and rate ratios for hospitalisations related to bacterial respiratory tract infections (2008-2017). Hospitalisation rate ratios (RRs) are plotted on a logarithmic scale and were calculated with individuals born at term (week 39–41) as references. The error bars represent the 95% confidence intervals (95% CI) for the RRs. The results are adjusted for year of birth, maternal age, parity, and sex.

**Part II: supplemental outcomes**

Supplemental figure 4. Estimated yearly rates (per 1,000 person-years) and rate ratios for hospitalisations related to any infectious diseases, excluding respiratory tract infections (2008-2017). Hospitalisation rate ratios (RRs) are plotted on a logarithmic scale and were calculated with individuals born at term (week 39–41) as references. The error bars represent the 95% confidence intervals (95% CI) for the RRs. The results are adjusted for year of birth, maternal age, parity, and sex.

**Part II: supplemental outcomes**

Supplemental figure 5. Estimated yearly rates (per 1,000 person-years) and rate ratios for hospitalisations related to viral infectious diseases, excluding respiratory tract infections (2008-2017). Hospitalisation rate ratios (RRs) are plotted on a logarithmic scale and were calculated with individuals born at term (week 39–41) as references. The error bars represent the 95% confidence intervals (95% CI) for the RRs. The results are adjusted for year of birth, maternal age, parity, and sex.

**Part II: supplemental outcomes**

Supplemental figure 6. Estimated yearly rates (per 1,000 person-years) and rate ratios for hospitalisations related to bacterial infectious diseases, excluding respiratory tract infections (2008-2017).  Hospitalisation rate ratios (RRs) are plotted on a logarithmic scale and were calculated with individuals born at term (week 39–41) as references. The error bars represent the 95% confidence intervals (95% CI) for the RRs. The results are adjusted for year of birth, maternal age, parity, and sex.

**Part III: additional analyses on any infections**

Supplemental figure 7. Estimated yearly rates (per 1,000 person-years) and rate ratios for hospitalisations related to any infectious diseases, adjusted for birth weight SD-score (2008-2017). Hospitalisation rate ratios (RRs) are plotted on a logarithmic scale and were calculated with individuals born at term (week 39–41) as references. The error bars represent the 95% confidence intervals (95% CI) for the RRs. The results are also adjusted for year of birth, maternal age, parity, and sex.

**Part III: additional analyses on any infections**

Supplemental figure 8. Estimated yearly rates (per 1,000 person-years) and rate ratios for hospitalisations related to any infectious diseases, adjusted for maternal education (2008-2017). Excluding 118,394 individuals with missing information on maternal educational level. Hospitalisation rate ratios (RRs) are plotted on a logarithmic scale and were calculated with individuals born at term (week 39–41) as references. The error bars represent the 95% confidence intervals (95% CI) for the RRs. The results are also adjusted for year of birth, maternal age, parity, and sex.

**Part III: additional analyses on any infections**

Supplemental figure 9. Estimated yearly rates (per 1,000 person-years) and rate ratios for hospitalisations related to any infectious diseases, adjusted for maternal country of birth (2008-2017). Excluding 308,148 individuals with missing information on maternal country of birth. Hospitalisation rate ratios (RRs) are plotted on a logarithmic scale and were calculated with individuals born at term (week 39–41) as references. The error bars represent the 95% confidence intervals (95% CI) for the RRs. The results are also adjusted for year of birth, maternal age, parity, and sex.

**Part III: additional analyses on any infections**

Supplemental figure 10. Estimated yearly rates (per 1,000 person-years) and rate ratios for hospitalisations related to any infectious diseases in 2008–2017, adjusted for birth month in two categories of Spring–Summer (March–August) and Fall–Winter (September–February) months, year of birth, maternal age, parity, and sex. Hospitalisation rate ratios (RRs) are plotted on a logarithmic scale and were calculated with individuals born at term (week 39–41) as references. The error bars represent the 95% confidence intervals (95% CI) for the RRs.

**Part III: additional analyses on any infections**

Supplemental Figure 11. Estimated yearly rates (per 1,000 person-years) and rate ratios for hospitalisations related to any infectious diseases, excluding 84,226 individuals with congenital malformations (2008-2017).  Hospitalisation rate ratios (RRs) are plotted on a logarithmic scale and were calculated with individuals born at term (week 39–41) as references. The error bars represent the 95% confidence intervals (95% CI) for the RRs. The results are adjusted for year of birth, maternal age, parity, and sex.

**Part III: additional analyses on any infections**

Supplemental figure 12. Estimated yearly rates (per 1,000 person-years) and rate ratios for hospitalisations related to any infectious diseases, excluding 4,375 individuals with any CP/severe disability (contact with hospital with diagnosis between 2008 and 2017). Hospitalisation rate ratios (RRs) are plotted on a logarithmic scale and were calculated with individuals born at term (week 39–41) as references. The error bars represent the 95% confidence intervals (95% CI) for the RRs. The results are adjusted for year of birth, maternal age, parity, and sex.

**Part III: additional analyses on any infections**

Supplemental figure 13. Estimated yearly rates (per 1,000 person-years) and rate ratios for hospitalisations related to any infectious diseases, excluding 75,519 multiples (2008-2017). Hospitalisation rate ratios (RRs) are plotted on a logarithmic scale and were calculated with individuals born at term (week 39–41) as references. The error bars represent the 95% confidence intervals (95% CI) for the RRs. The results are adjusted for year of birth, maternal age, parity, and sex.

**Part IV: overview**


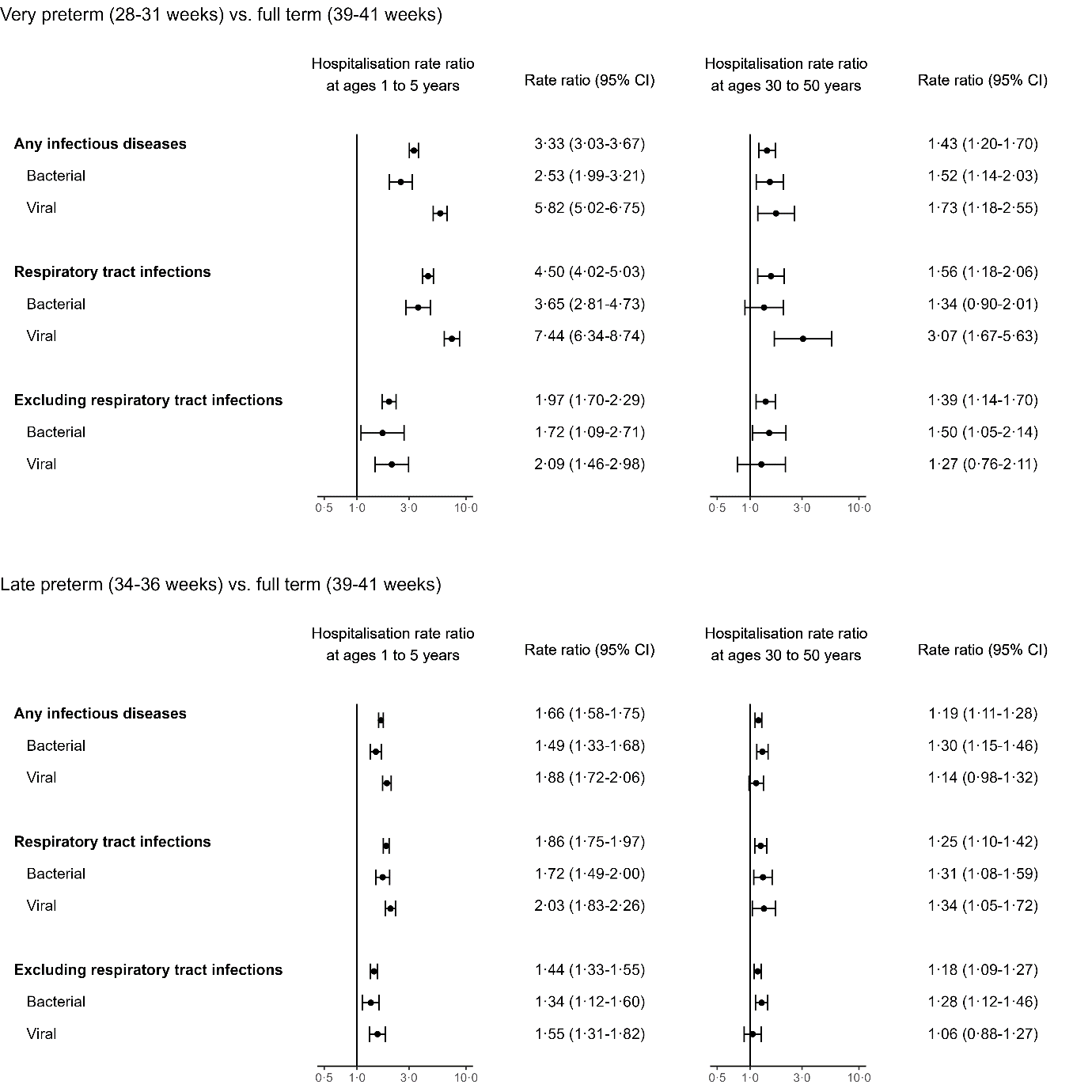


Supplemental figure 14. Estimated hospitalisation rate ratios for any infectious diseases, respiratory tract infections and any infectious diseases excluding respiratory tract diseases (2008-2017). Hospitalisation rate ratios are plotted on a logarithmic scale and were calculated with individuals born at term (week 39–41) as references. The error bars represent the 95% confidence intervals (95% CI). The results are also adjusted for year of birth, maternal age, parity, and sex.
